# Localising ingested metallic foreign bodies to guide surgical planning: A novel use for the CT metal artefact reduction algorithm

**DOI:** 10.1259/bjrcr.20200189

**Published:** 2021-06-16

**Authors:** Peter Abernethy, Neil G McIntyre, Alexander Sanchez-Cabello, Ross Kruger, Dushyant Shetty

**Affiliations:** 1 Royal Cornwall Hospital, Truro, United Kingdom; 2 Peninsula Radiology Academy, Plymouth, United Kingdom

## Abstract

We present the case of a 20-year-old female patient who presented following ingestion of multiple button magnets. She remained clinically well however serial abdominal radiographs demonstrated the magnets were not passing through the gastrointestinal tract and a CT was, therefore, performed for further assessment and to aid surgical planning. Artefact from the magnets made interpretation of the CT challenging. The use of a Metal Artefact Reduction (MAR) algorithm, however, enabled accurate localisation of the magnets thus guiding subsequent surgical intervention. Whilst MAR algorithms are usually used in the assessment of iatrogenic metallic devices (*e.g.,* joint prostheses), this case demonstrates an example of their potential wider use.

## Clinical presentation

A 20-year-old female known to the community psychiatry team presented to the general surgeons two days after ingestion of multiple button magnets. Her only symptom was that of nausea and her physical examination was unremarkable.

## Investigations/Imaging Findings

**Figure 1.**
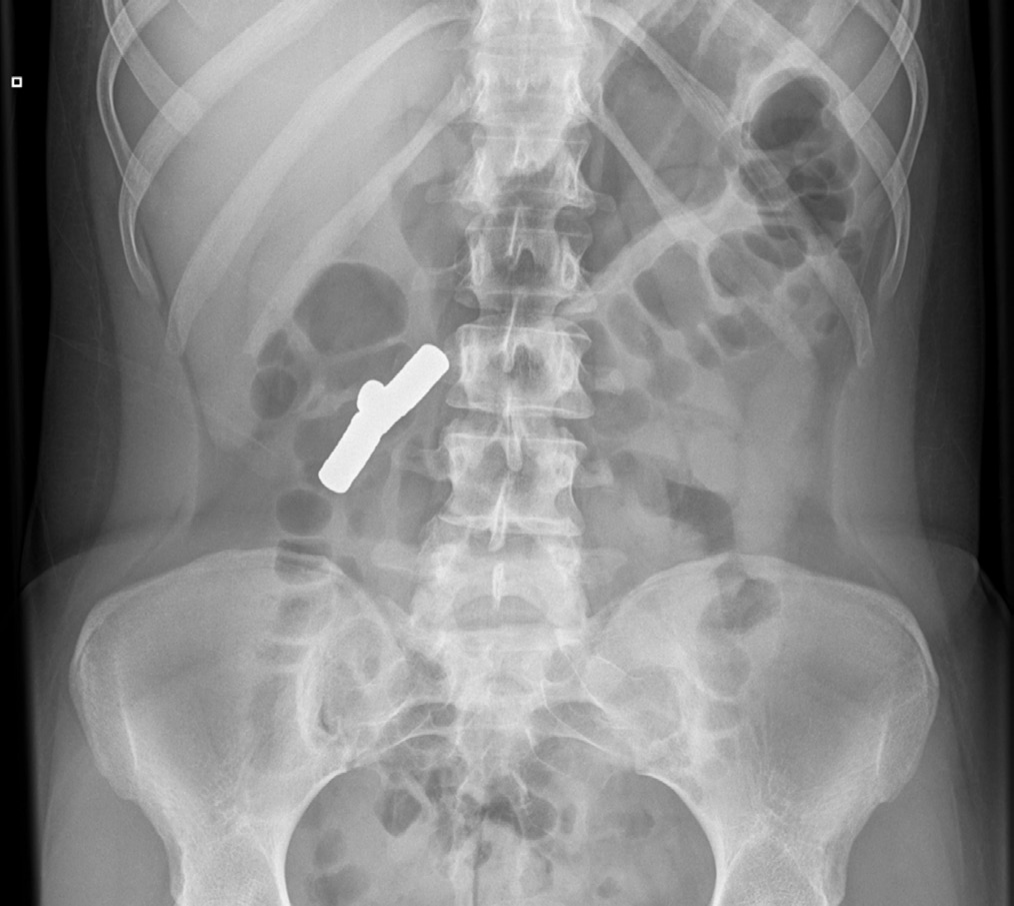
Abdominal radiograph showing the aggregation of button magnets in the right upper quadrant.

Her white cell count (WCC) and C-reactive protein (CRP) were not elevated. An initial abdominal plain radiograph demonstrated well-defined metallic opacities in the right upper quadrant with no bowel dilatation or evidence of perforation. Clinical stability and the lack of bowel distension lead to a conservative approach, hoping for physiological passage of the magnets (Figure 1).

Serial abdominal plain films over the following days, however, showed an unchanged position of the magnets. The patient remained clinically well and blood tests were normal. Three days after admission, the lack of magnet transit on serial abdominal radiographs resulted in a CT of the abdomen and pelvis for accurate localisation (Imaging was performed on a Toshiba Aquilion ONE 128 multislice system.) This was done without i.v. contrast as there was no clinical suspicion of bowel ischaemia. The need for endoscopic/operative intervention had already made prior to the CT scan request. No positive oral contrast was given to avoid obscuring the magnets with the intraluminal increased attenuation. The primary aim of the scan was anatomical localisation of the magnets.

The images demonstrated the magnets in the right upper quadrant of the abdomen. Assessment of their precise anatomical location was restricted due to significant artefact from the metallic foreign bodies, which also limited the evaluation for local complications.

**Figure 2.**
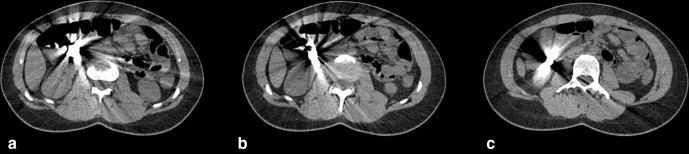
a,b,c: Sequential non-contrast axial CT images, demonstrating the aggregation of button magnets. No metal reduction artefact applied.

**Figure 3.**
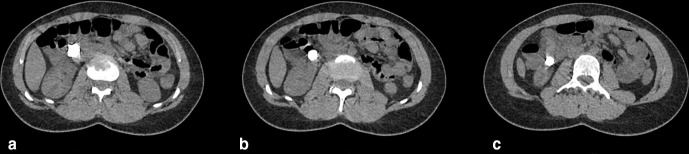
a,b,c: Sequential non-contrast axial CT images, demonstrating the aggregation of button magnets. Metal reduction artefact applied.

**Figure 4.**
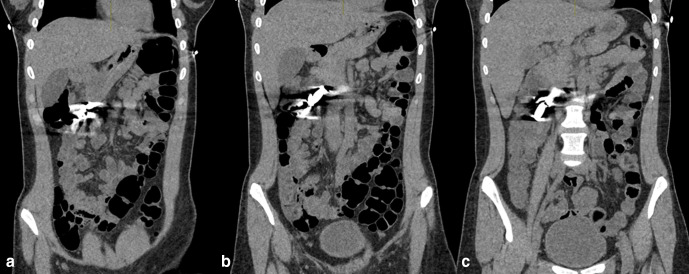
a,b,c: Sequential non-contrast coronal CT images, demonstrating the aggregation of button magnets. No metal reduction artefact applied.

**Figure 5.**
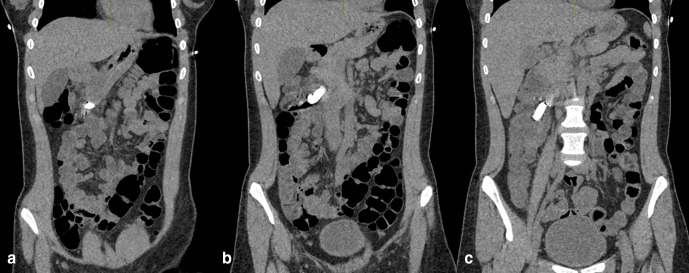
a,b,c: Sequential non-contrast coronal CT images, demonstrating the aggregation of button magnets. Metal reduction artefact applied.

**Figure 6.**
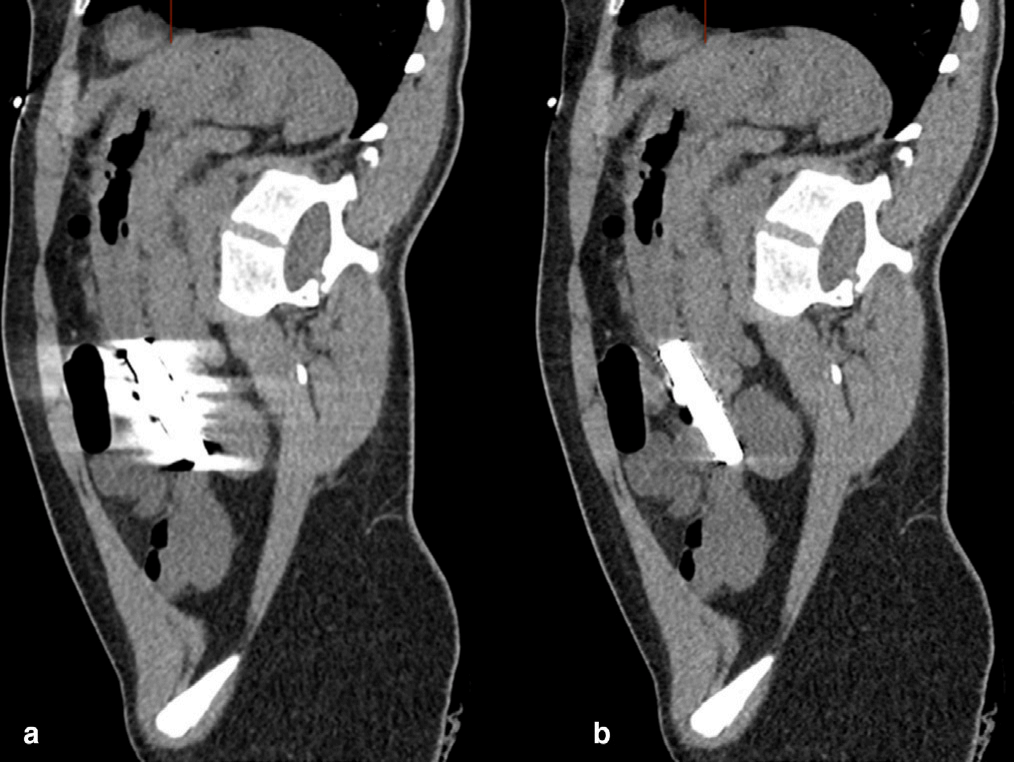
a,b: Sagittal oblique MPR CT image in the plane of the magnets, demonstrating the aggregation of button magnets. (**a**) No metal reduction artefact applied. (**b**) Metal reduction artefact applied.

With subsequent reconstruction of the image data using an MAR algorithm, it then became evident that there were magnets present in the gastric antrum and in D2/D3, which had attracted to one another. The magnets had created apposition between gastric and duodenal mucosal surfaces (Figures 2–6).

## Management

Oesophagogastroduodenoscopy (OGD) was performed that demonstrated one magnet in the gastric antrum with adjacent mucosal erosion. Intubation of the duodenum showed a column of magnets lying transversely across D2/D3 with erosion of the duodenum. Subsequent laparotomy revealed a column of 18 button magnets lying across the gastric antrum, duodenum and transverse colon. This resulted in fistulation across these three structures through large pressure defects, which were all repaired.

Post-operatively the patient made a good recovery and was discharged home with community psychiatric follow-up.

## Discussion

As demonstrated in this case report, ingestion of multiple magnets can cause significant morbidity including gastrointestinal necrosis, perforation, fistulation and obstruction. It is a presentation more typically seen in the paediatric population with one retrospective analysis finding that 69.7% of children require surgical intervention after ingesting multiple rare-earth magnets.^1^ Intentional foreign body ingestion in the adult population is often associated with psychiatric illness, for instance personality disorders and pica.^2^ In this population, it can present a diagnostic challenge as the history may be concealed and there is often no clinical evidence of foreign body ingestion until a complication has occurred.

CT imaging is considered by many as the gold standard for determining the location of foreign bodies in the context of ingestion and penetrating trauma (in the acute and postmortem setting). CT is thought to be particularly useful when assessing foreign bodies of unknown composition..^3,4^ CT is also superior to plain radiography and ultrasound at detecting foreign bodies in gas-filled structures, which proved particularly useful in this case.^5^

Metallic foreign bodies and implants are problematic when imaging with CT as the artefacts they cause reduce the image quality of adjacent tissues and also limit assessment of the metallic objects themselves. Such artefacts are typically due to either photon starvation or beam hardening. With diagnostic imaging, the primary contributing factor for both of these is the photoelectric effect. This is predominately determined by the density (or atomic number) of the material being imaged.

Photon starvation typically occurs when there is severe attenuation of the beam to the point that insufficient photons reach the detector. The reconstruction of this limited data in the final image results in streaks of black and white centring on the causative structure, in this case the ingested magnets.

Beam hardening is a consequence of the X-ray beam being polychromatic in energy and preferential attenuation of lower energy photons. The result of this is that a beam that passes through a substance such as metal is “hardened”. As CT reconstruction relies on data acquired from multiple projections, for which the hardening effect is likely to be different, the result is inconsistent data acquisition and a resultant artefact on the images. In practice, this often appears as areas of low density between two denser structures (*e.g.,* between metallic hip replacements) and can also be seen as “cupping” artefact when scanning phantoms for quality assurance.^6,7^

There are several ways in which the above-described artefacts can be mitigated in order to decrease the impact on the final reconstructed image and improve the diagnostic adequacy of the scan.

With regard to beam hardening, tilting of the gantry, correct selection of the most appropriate field of view, and use of the appropriate bowtie filter can all be used

. More advanced techniques have also been introduced that take advantage of dual-energy CT. Unfortunately, most of these require not only a compatible system but also, with the exception of dual layer detection systems, a prospective choice to use the dual energy analysis.^7,8^

The simplest ways to reduce photon starvation are to increase the number of photons generated, by using a higher tube current, or increasing the energy of the beam by using a higher peak voltage. However, both of these will have a limited affect with regard to metallic artefact and result in higher doses to the patient. In order to mitigate this, many CT scanner manufacturers have developed projection-based MAR algorithms like that utilised in this case. These typically operate by isolating voxels, which exceed a set Hounsfield unit level to identify a metallic structure. From this, it is possible to detect artefactual data which is then subtracted from the final image and replaced with interpolated and back-projected data resulting in a corrected image. These can often be applied retrospectively once a CT dataset has been acquired.

There are some pitfalls associated with the use of MAR algorithms. Metallic implants themselves can be interpreted as part of the artefact and, thereforecan be partially or completely removed on adjusted images. Further errors can also be introduced during the correction step when the corrupted data are estimated by the algorithm. As such they are associated with their own range of artefacts. Therefore, where it has been used, images with and without the MAR algorithm applied should be reviewed in conjunction in order to provide the most accurate information.

Most available literature has evaluated the use of MAR algorithms in assisting the interpretation of pathology close to iatrogenic metal objects such as orthopaedic implants, most commonly constructed from titanium, and embolisation coils, commonly platinum-based.^8,9^ To the authors’ knowledge, there is no medical research or case reports on the use of MAR algorithms to improve imaging quality around an ingested metallic foreign body.

## Learning points

Use of CT MAR algorithms can improve anatomical localisation of ingested metallic foreign bodies to help further management. In most cases, it can be applied retrospectively.

MAR techniques may be useful in other clinical settings such as penetrating projectile trauma and retained metallic surgical material although should be used with caution and always be reviewed with the original images.

Ingested magnets require early investigation and timely invasive management to prevent complications.
